# Exploring the quality of life of caregivers of children with sickle cell disease in a secondary health facility in Ghana

**DOI:** 10.4314/gmj.v59i4.6

**Published:** 2025-12

**Authors:** Samuel T Salamat, Haruna Mahama, John Asabi, Lydia Asante

**Affiliations:** 1 Holy Family Hospital, P.O. Box 21, Berekum, Ghana; 2 St. Theresa's Hospital, P.O. Box 15, Nandom, Ghana; 3 Catholic University of Ghana, P.O. Box 363, Fiapre -Sunyani, Ghana

**Keywords:** Sickle cell disease, Quality of life, Caregivers, Children

## Abstract

**Objective:**

To explore the caregiving burden of caregivers of children with Sickle Cell Disease (SCD) to provide a foundation for policy interventions

**Design:**

A qualitative study based on convenience sampling

**Setting:**

The Sickle Cell Clinic of the Paediatric Department of a secondary health facility

**Participants:**

Primary caregivers of children with SCD who had been attending the clinic for at least one year were included. Caregivers whose children had another chronic illness were excluded to avoid confounding. Eleven care-givers participated in the study.

**Interventions:**

No intervention

**Main outcome measures:**

Caregiving burden among participants caring for children with Sickle Cell Disease (SCD).

**Results:**

The four main themes that emerged from the study were the financial, psychological, social, and physical burdens of caregiving. The financial and psychological burdens were noted to have the most substantial impact on their quality of life.

**Conclusion:**

Addressing the needs of caregivers of children with SCD goes beyond financial support. Providing psychological interventions is crucial to alleviating the emotional burden of the disease on caregivers. The study revealed the multifaceted challenges faced by caregivers; hence, the need for a holistic approach to support families affected by SCD in Ghana.

**Funding:**

None declared

## Introduction

Sickle cell disease (SCD) is the most common haemoglobinopathy affecting an estimated 4.4 million people worldwide, with 80% living in sub-Saharan Africa. Available data suggest that about 43 million people have the sickle cell trait.^1^,^2^ Worldwide, more than 300,000 babies are born with the disease annually, with over 75% in sub-Saharan Africa.^3^,^4^ The incidence in Cameroon, DR Congo, Gabon and Ghana ranges from 20-30% and may be as high as 45% in some parts of Uganda.^5^ It is estimated that 2% of babies born in Ghana (20 for every 1000 live births) have SCD. The frequency of the sickle cell trait ranges from 2% to 30% among the general population, with one in four of the population carrying either the haemoglobin S or C trait.^6^–^9^ Among cases of SCDs in sub-Saharan Africa, 50-80% are estimated to die from complications of the disease before their fifth birthday, which has been largely attributed to late diagnosis and therapeutic intervention.^10^ However, in developed countries, early diagnosis using routine neonatal screening programmes and comprehensive patient care interventions has resulted in most infants with the disease surviving into adulthood.^11^

SCD is a chronic disease characterised by recurrent haemolytic anaemia, acute crises due to occlusion of the blood vessels and recurrent infections.^12^ These sickling crises include acute vaso-occlusive crises, which commonly present as bone pain; haemolytic crises, which present as worsening anaemia and jaundice; splenic sequestration, which clinically will be evident as worsening splenomegaly and anaemia; and finally aplastic crises, which occur following infection with the Parvovirus B19.^13^ Extremes of temperature, infections, dehydration, and hypoxia trigger these acute sickling crises.^7^

Complications of the disease result in frequent hospital visits for prolonged periods, to receive often costly treatments. The disease, hence, adversely affects the quality of life of both the patient and the caregiver due to the high social and economic impact. Owing to its impact, the UN General Assembly in 2008 declared it a public health problem.^10^

Caregiving burden is defined by Zarit et al 14 as “the extent to which caregivers perceive the adverse effect that caregiving has on their emotional, social, financial and physical functioning. This definition shows the multifaceted challenges experienced by caregivers.^15^ The care-giving burden correlates with the Quality of Life (QOL) of the caregiver.^16^ The WHO Quality of Life Group (WHOQOL) in 1995 defined Quality of life (QOL) as an “individual's perception of their position in life in the context of the culture and value systems in which they live and in relation to their goals, expectations, standards and concerns”.^17^ QOL of both patient and caregiver is a key component of the care of children with chronic diseases such as SCD.^18^ The long-term irreversible organ complications of the disease and the low socio-economic state of most caregivers place a major strain on the patients and caregivers alike.^19^ Therefore, children with SCD have significantly impaired health-related quality of life with detrimental effects on the family income and associated poor quality of life among caregivers.^20^

Activities related to caregiving, such as administering medications and taking the patient to the hospital for periodic visits or hospitalisation, adversely affect the caregiver's physical health.^21^ A study done in Nigeria discovered that mothers of SCD patients suffered significant psychosocial impairment and that attention needed to be paid to this need of such mothers since this potentially affected their ability to care for these children.^22^ Social isolation and disruption in social activities were identified as components of the social burden of caregiving for children with chronic illnesses.^23^ The cost of periodic hospital visits, frequent hospitalisations, and the purchase of medications such as hydroxyurea pose an enormous financial burden on the family budget.^24^,^25^

The overall well-being and quality of care given to children with SCD are directly influenced by the quality of life of their caregivers. As a result, the health needs of such caregivers should be of public health importance, requiring immediate intervention from health workers and policymakers.^26^ Assessing the quality of life of such caregivers will help determine life-changing medical interventions, provide clinicians with insight into the perceived burden of care, and help allocate necessary resources within the health delivery system to meet the needs of such caregivers.^16^

Despite the high prevalence of the disease in Ghana, there is a paucity of published research on the quality of life of caregivers, particularly in peri-urban areas. The study sought to explore the perceived caregiving burden in such an area and to provide a foundation for effective intervention strategies for policymakers.

## Methods

### Study design and settings

An exploratory design with a phenomenological qualitative approach was used to study the QOL of caregivers of children with SCD in the Berekum Municipality. This study design was deemed appropriate for the research as it enabled the researchers to gain more insight into the perceptions, feelings, and experiences of caregivers of children with SCD. The write-up of the study was guided by the Consolidated Criteria for Reporting Qualitative Research (COREQ).

The study was conducted at the Sickle Cell Clinic of the Paediatric Department, Holy Family Hospital in the Berekum Municipality. Holy Family Hospital is an accredited secondary health facility that serves as a referral centre for the Municipality and neighbouring districts. The hospital has a bed capacity of 162 with an average daily outpatient attendance of 350 patients. The Sickle Cell Clinic of the Paediatric unit was established in January 2020 and had 100 patients registered as of July 2022. The clinic maintains a record of 92 caregivers of children with SCD, making it a suitable site for the study.

### Data collection

A semi-structured interview guide, designed in relation to the objectives of the study, was used for data collection. Primary caregivers with at least one child with SCD and who had been attending the clinic for at least one year were included. A caregiver whose child had another chronic illness in addition to SCD was excluded from the study, as another chronic illness would be a confounder to the perceived caregiving burden of the caregiver.

Data collection was done from 22^nd^ August to 9^th^ September 2022. The interview guide was sectioned into two parts (A and B). Part A of the interview guide was designed to collect demographic data from participants. Part B collected data on the burden of caregiving. Face-to-face in-depth interviews were conducted with participants who consented to the study. In addition to the interviews, audiotape recordings of all interviews were made, while field notes were also taken during interviews to capture the emotions expressed by participants. Apart from one participant, in which the interview was conducted in English, all others were in the Twi language.

All the interviews were conducted at the Sickle Cell Clinic, except for one, which was done at the paediatric ward. Interviews were started by asking participants to give their general experience with caring for a child with SCD. Subsequent questions then focused on the objectives of the study. Probing was used to elicit further details where necessary. Interview sessions lasted between 18 and 35 minutes. An audiotape recording from each interview was transcribed within 24 hours of the interview.

### Data analysis

An inductive qualitative approach was used to analyse the data using the computer-based software NVivo.12 (http://www.qsrinternational.com). Prior to the analysis, the ten recorded Twi interviews were translated into English and transcribed verbatim by the first author. Although a language expert was not employed, two nurses who are highly proficient in the Twi language also carried out the translation and transcription. Each nurse independently translated and transcribed the audiotapes, and their transcripts were compared. No significant discrepancies in meaning were noted between the nurses' transcripts and those of the first author, and hence no corrections were made. Transcripts were read several times for familiarity and to identify similar patterns. Coding was done using NVivo.12 software. The generated codes were then grouped into themes and sub-themes. The identified themes were then reviewed, and where necessary, modifications were made to them. In-depth descriptions of the themes and sub-themes were done and supported with quotes from participants.

### Rigour

The framework by Guba and Lincoln^27^ was used to ensure the credibility, transferability, dependability, and confirmability of the study. Credibility was achieved by reading transcripts to participants to ascertain that they represented what was said during the interviews. Transferability, on the other hand, was achieved by providing a comprehensive description of the study setting, and the study was subjected to critical review by the fourth author, an experienced researcher and academic supervisor, to ensure dependability. Also, confirmability was achieved by making the data available for verification and authentication by the third and fourth co-authors, who reviewed the study results.

### Ethical consideration

Ethical clearance (study ID number: KHRCIEC/2022-210) was obtained from the Kintampo Health Research Centre Institutional Review Board before commencement of the study. A letter was sent to the Management of Holy Family Hospital for permission to use the facility as a study site. Participants were informed about the study, and those who consented were scheduled for an interview at their convenience. Written consent was obtained before the start of the interview by having participants either sign or thumbprint the consent form. They were informed that participation in the study was voluntary. All information collected from the survey was kept confidential and safe. There were no direct benefits for participants.

## Results

### Demographic characteristics of participants

Eleven (11) participants were interviewed until data saturation was reached, with nine (9) being females. Most of the participants (9) were between the ages of thirty (30) and forty (40), with eight (8) of them married. In terms of their educational attainment and employment, five (5) had attained basic level education, and many of them (7) were self-employed. All but one participant practised Christianity. Most participants (8) had one child with SCD, with most of these children (9) having SCD phenotype SS.

### Themes

Four themes emerged after analysing the data using NVivo 12. These themes were financial, psychological, physical, and social burdens. Various subthemes were identified and classified under the themes, as shown in Table 2.

**Table 2:** Themes and Subthemes

Themes	Subthemes
**Financial burden**	Purchasing of medications Cost of hospital visits/hospitalisation
**Psychological burden**	Fear Regret Worry Stigma
**Physical burden**	Inadequate sleep
**Social burden**	

Source: Authors construct (2022)

### Financial burden

The main sub-themes that emerged from this theme were the purchasing of medications and the cost of hospital visits/hospitalisation.

### Purchasing of medications

Most respondents reported that purchasing medications posed a significant financial burden. They expressed how the disease necessitated the constant purchase of medications, which negatively impacted their finances, as per the quotes below.

*“Her condition has put a financial burden on me, especially in buying drugs for her and providing a balanced diet for her”* (P2)

*What I will say is that it is a disease that has put some financial burden on me in terms of buying drugs and when they fall ill, but I will say it has not drastically affected my finances. The NHIS has helped to offset some of the financial burden of the disease.”* (P8)

*“….*. *I must borrow money to buy drugs, and sometimes, by the time we are discharged from the hospital, there is no money to buy food. The little I get from my petty trading, I use it for his hospital bills”* (P6)

### Cost of hospital visits/hospitalisation

This sub-theme highlights the financial burden experienced by respondents due to frequent hospital visits and hospitalisation. This was reflected in the following statements:

*“For now, the main effect on my life is the financial burden. I must spend money to buy his drugs, and if he is admitted, we incur a lot of debt.”* (P11)

*“It has affected me greatly; I need to get money to buy drugs, for hospital admissions, and for hospital reviews* (P1)

*“Yes, the drugs and hospital fees, but like I said, I am very grateful to those who introduced the health insurance scheme. I must commend them because it has helped me so much.”* (P3)

*“In what he eats, I need to also save money to buy drugs for him and to come for monthly reviews. Sometimes I save money to use for my petty trading, only for that money to be used for his hospital expenses.”* (P4)

*“You know, there are times when he falls ill when we do not have money. When that happens, it becomes difficult for us, but my husband, being the head of the family, must manage and find the money for his medical bills”* (P9)

### Psychological burden experienced by caregivers of children with SCD

This theme comprised four sub-themes: Fear, Regret, Worry, and Stigma.

### Fear

All respondents feared that their children would die young from the disease. This fear was expressed in comments such as:

*“…*. *I worried about the perception out there that such children do not live long and can die at any time….”* (P7)

*“There is a perception out there that such children do not live up to even ten years old; a lady once told me that I should not even count him as one of my children because he can die at any time”* (P11)

*“For me, my main worry was that my child, whom I have given birth to, can die at any time; his father is so fond of him and so whenever he falls ill, he gets so worried, and that worries me as well.”* (P5)

*“Yes, very much, I worry about him a lot; people say children with this condition do not live long and can die at any time ….”* (P1)

### Regret

Three (3) participants, in addition to the fear of their children dying young, also expressed regrets about giving birth to their children.

*“…*. *if I had known things would turn out this way, I would not have given birth to him because every time he falls sick, I become worried, coupled with the frequent hospital visits.”* (P4)

*“… I would not have given birth to him if I had known he would be born with this condition. It has really affected my life. If not for this child, I would have travelled abroad for greener pastures….”* (P1)

*“I sometimes regret giving birth to him, but I encourage myself knowing that it is God who gave him to me”* (P9)

### Worry

Some respondents were worried about the impact of the disease on the children and their families. This was expressed as follows:

*“Yes, sometimes when I am there, I will be thinking of who will take care of my children or who will be close to my children the way I am. Some of my classmates are passing on, and I know the one who brought me into this world can call me at any time, but my wish is that I am personally around near my children for most of their lives until they are completely independent. Sometimes when I am there, I begin to ask myself questions that if I am not there today, who will care for them as well as I do”* (P3)

*“Sometimes I worry that if I do not have money and he gets ill and must be admitted, that may also be a problem. Secondly, I worry that in the future, when he is grown and wants to get married, and he meets someone that he loves, the disease may be a hindrance to them getting married.”* (P7)

*“I have been worried about the condition ever since he was diagnosed. It affects his schooling. During the academic year, he often has acute crises and is unable to go to school. Because of this frequent absenteeism from school, he has had to repeat the same class several times.”* (P6)

*“I worry about the financial impact of the disease on my husband. I am a petty trader, and because the disease is in my family and I have the disease, sometimes I get a bone crisis. Because of the financial burden and the pressure it puts on my husband, he is unhappy and often verbally abuses me. I do not have any happiness in my marriage, and if I had my own way, I would have asked for a divorce. {Respondent breaks down and starts weeping}* (P6)

*“The way he cries when he gets bone crises makes me pity him, and that gets me worried.”* (P11)

### Stigma

Two respondents were concerned about stigma due to the disease. One cited an instance of stigmatisation, and the other about potential stigma by friends.

*“… recently, one of my neighbours told his children not to eat with him because they would contract the disease if they ate with him. I was so sad when I heard it. I had to explain to those children that this is a disease of the blood that you are born with and cannot be contracted by eating with a person who has the disease.”* (P4)

*“Yes, that is the more reason why I do not tell my friends about his condition. Some of them will not want him to play with their children if they get to know of his condition.”* (P9)

### Social burden of caring for children with SCD on the QoL of caregivers

Most participants (8) said the condition generally had no social burden on them. They said the disease did not have any adverse effect on their social interactions, and they can attend any social gatherings except in cases when their children are acutely ill and may have to be admitted or cared for at home.

*“I am not an outgoing person, but his condition does not affect how I interact with others. My friends sometimes ask me why my child is always sick, but when I explain to them, they understand and encourage me. No one stigmatises me because of his illness”*. (P1)

*“It does not really affect my social life; I can go wherever I want to go and interact with others”* (P4)

*“As for social gatherings, I can go, but if he falls acutely ill for that one, I have to stay and take care of him”*. (P9)

*“If he is not acutely ill, I can attend social gatherings such as funerals; I leave him in the care of my elder sister or my mother, but when he is acutely ill, I am not able to go.”* (P11)

### Physical burden of caregiving on the QoL of caregivers

Concerning the physical burden of caring for a child with SCD, some participants (7) said the disease hurt their physical health. The main effect was inadequate sleep, which was experienced especially during episodes of acute crises.

*“I usually carry him at my back to my workplace and to the hospital, but I won't say that has affected my physical health. I do not have any discomfort or back pain from carrying him. Caring for him has, however, affected my sleep pattern. I sleep late and must wake up early because of him”* (P1)

*“It does not really affect my health except when she has an acute crisis, during which time it affects my sleep and everything that I do” (P2)*

*I am not able to sleep, especially when he has an acute crisis, but even when he is not acutely ill, when I sleep and wake up in the night, I am no longer able to go back to sleep.”* (P11)

*“It is the constant worrying that gives me sleepless nights. I worry so much that sometimes I develop a headache. Sometimes I feel there is a heavy burden on me, but I have no one to help me.”* (P6)

Four respondents, however, felt the disease had no adverse effect on their physical health. This was expressed in comments such as:

*“Not really, I used to worry, but in terms of my physical health, it doesn't affect me.”* (P4)

*“In terms of my physical health, it has no effect. It is only the little worry about the fact that he may die at any time, but that has not affected my physical health.”* (P8)

## Discussion

Caring for a child with SCD is a challenging experience for the caregiver. The activities of caregiving, such as administering routine medications, home care during periods of acute crisis, periodic hospital visits and hospitalisation are highly demanding for the caregiver and other family members. Caregiving burden correlates with the caregiver's quality of life, and this burden is categorised as financial, psychological, social, and physical. Findings from this study revealed that caregiving of children with SCD was associated with an immense financial burden on the caregivers. Participants stated they needed money to purchase medications such as Penicillin V and hydroxyurea, attend routine hospital visits, and hospitalisation for acute crisis, a finding similar to studies.^24^,^25^ Such expenditure poses an enormous financial challenge to the family purse. Time lost in caring for these children, especially during periods of acute crisis at the expense of engaging in profitable economic ventures, was also identified as a contributory factor to the financial burden of the disease on the caregivers. Some participants resorted to borrowing from friends to settle hospital expenses following hospitalisation of their children; others had to deny themselves some basic needs to meet the financial requirements of caring for their children. The financial burden of caring for a child with SCD from this current study is similar to several existing studies in Africa and other parts of the world.^19^–^21^,^28^–^30^. The National Health Insurance Scheme (NHIS) should expand its coverage to include medications for the acute and routine care of children with SCD. Although routine drugs such as folic acid and penicillin V were already covered, caregivers previously bore the full cost of hydroxyurea, a major disease-modifying drug, until its inclusion on the NHIS list in July 2022. Some essential medications for acute management, however, remain inadequately covered, adding to the financial burden on caregivers.

The care of a child with SCD was also found in this study to have an adverse psychological impact on caregivers. The fear of their children dying prematurely, regret, guilt, and worry were the negative emotional impacts of the disease, stated by the respondents. This negative psychological effect of regret, guilt, and worrying is consistent with previous studies.^19^,^21^,^28^,^31^ The chronic nature of the illness, the unpredictable nature of acute crisis, and the lack of attention paid to the psychological needs of these caregivers have contributed to the significant emotional burden posed by the disease on the caregivers. Stigmatisation of both the caregiver and the child was also identified in this study to contribute to the emotional burden of the disease. Previous qualitative studies in Kenya and Ghana also reported similar findings of stigmatisation among mothers of children with SCD.^32^,^33^

The Paediatric Department of Holy Family Hospital should establish a social support group for caregivers of children with SCD attending the clinic, providing both emotional and, where possible, financial assistance. Additionally, professional psychological support should be integrated into routine care, beginning at diagnosis, during hospitalisations, and at regular intervals, ideally at least quarterly.

The social burden of caring for a child with SCD from the study was noted to have minimal effect on the care-givers. The day-to-day activities of most respondents were generally not affected. Most participants were able to attend social gatherings such as funerals and wedding ceremonies, except in instances of hospitalisation of their children. This finding is in contrast to previous studies, which reported that the routine social activities of care-givers of children with chronic illness were negatively affected.^23^,^34^ This minimal social burden of the disease may be due to support from other family members in caring for the children, thereby allowing the primary caregiver to attend to other social responsibilities. This task sharing by spouses and other close family members is a valuable resource in a peri-urban community such as Berekum, where the extended family system is still practised with close family members providing the needed assistance, especially in the care of children in the family. With the present modernization of the Ghanaian society with the focus now on the nuclear family system, especially in the urban settings, this communal care practice by members of the extended family system continues to be an asset in Berekum as this serves as a robust support system for affected families and parents, which in the long term can foster resilience and positivity in the parents.

Participants in this study did not think caring for their children had any major untoward effect on their physical health. Nevertheless, the main physical burden mentioned by participants was inadequate sleep during periods of acute crisis or from worrying about the disease. This finding is consistent with a study done in Amsterdam, Netherlands, to assess the quality of life of caregivers of children with SCD.^19^ Since the majority of the respondents in this study were young, in their thirties and forties, with no known chronic illnesses, it can be assumed that this peculiar demographic characteristic may have contributed to the caregiving burden not having a significant effect on their physical health. Future studies could focus on parents with children in their teens or older adults who, due to the nature of the disease, continue to be dependent on their parents. This will give an insight into how the advancement in age in both parents and children would impact the caregiving burden associated with SCD.

Finally, future research should employ quantitative or mixed methods designs and involve multiple healthcare facilities managing children with SCD to generate broader insights into the caregiving burden and inform more comprehensive interventions.

### Limitations of the study

Since the interview guide was in English, translation for participants who did not understand English may have introduced interpretation errors, potentially affecting the accuracy of the information obtained. In addition, transcribing Twi audio recordings into English may have affected the accuracy of participants' responses. The findings from this research can also not be generalised to the whole population as the study used a qualitative approach with a small sample size and a convenience sampling technique.

## Conclusion

Caring for children with SCD is associated with stressful activities such as consistent drug administration, regular hospital visits, ensuring adequate nutrition, preventing, and managing acute painful crises. These burdensome activities of caregiving pose enormous financial challenges. The care of such children is also associated with psychosocial burden, such as depression, anxiety, feelings of guilt and regret, worrying, stigmatisation and disruption of the family unit. These caregiving activities, coupled with associated challenges, have been shown to adversely affect caregivers' quality of life.

Regarding the various domains of caregiving burden, financial and psychological burden had a major impact on the quality of life of caregivers. The social burden of the disease was minimal, with inadequate sleep being the main physical effect of the disease on caregivers.

## Figures and Tables

**Table 1:** Demographic characteristics of participants

Variable	n (%)
**Age**	
**Mean age ±SD**	42±2 Years
**Gender**	
**Female**	9 (81.8)
**Male**	2 (18.2)
**Occupation**	
**Self-employed**	7 (63.6)
* Others	4 (36.4)
**Level of education**	
**Basic**	5 (45.5)
**Secondary**	2 (18.2)
**Tertiary**	4 (36.4)
**Marital status**	
**Married**	8 (72.7)
**Single**	2 (18.2)
**Divorced**	1 (9.1)
**Number of children with SCD**	
**One**	8 (72.7)
**Two**	3 (27.3)
**Religion**	
**Christianity**	10 (90.9)
**Islam**	1 (9.1)
**Phenotype of children**	
**SS**	9 (81.8)
**SC**	2 (18.2)

* Others: Midwife, Retired public servant, Typist, Makeup Apprentice
